# Skeletal growth in class II malocclusion from childhood to adolescence: does the profile straighten?

**DOI:** 10.1186/s40510-020-00313-9

**Published:** 2020-05-18

**Authors:** Matoula Taloumtzi, Melisa Padashi-Fard, Nikolaos Pandis, Padhraig S. Fleming

**Affiliations:** 1 Department of Oral and Maxillofacial Surgery, Barts and the London School of Medicine and Dentistry, London, UK; 2 Community Dental Service, Barts and the London School of Medicine and Dentistry, London, UK; 3 Department of Orthodontics, Dental School, Medical Faculty, University of Bern, Bern, Switzerland; 4 Private practice, Corfu, Greece; 5 Department of Orthodontics, Centre for Oral Bioengineering, Barts and the London School of Medicine and Dentistry, Queen Mary University of London, E1 2AD, London, UK

**Keywords:** Class II, Adolescent growth, Profile, Overjet

## Abstract

**Background:**

There is relatively little appreciation of the changes in maxillary-mandibular relationships occurring during adolescence among subjects with normal and increased overjet. The aim of this study was to assess differences in changes in maxillo-mandibular relationships during the adolescent growth period based on the presence of a normal (< 4 mm) or increased (> 4 mm) overjet in childhood. Our hypothesis was that there is no difference in the change of the A point, nasion, B point (ANB) angle during growth between these two overjet groups. Lateral cephalograms were obtained from 65 subjects taken from the American Association of Orthodontists Foundation (AAOF) Craniofacial Growth Legacy Collections Project. Cephalograms were obtained at ages 7–10 (T0) and 14–17 (T1) with allocation into two groups based on baseline overjet (> 4 mm: group 1, 2-4 mm: group 2). Random effects linear regression was used to account for multiple within -patient measurements with dependent variables including antero-posterior skeletal pattern (based on sella, nasion, A point (SNA); sella, nasion, B point (SNB); and ANB angles).

**Results:**

We included a similar number of males (*n* = 34; 52.3%) and females (*n* = 31; 47.7%). The mean ANB was higher at baseline in group 1 (5.42, SD 2.16°) than in group 2 (3.08, SD 1.91°). The hypothesis was rejected as the ANB angle reduced by 1.92° more in the larger overjet group with the association being statistically significant after accounting for age and gender (*P* < 0.001; 95% CI 1.06 to 2.77). No significant gender-related effect (*P* = 0.624; 95% CI − 0.637 to 1.07) was observed overall. However, there was no significant increase in SNA angle in the > 4 mm overjet group compared to the 2–4 mm group (0.857°, *P* = 0.271; 95% CI − 0.669 to 2.383). The SNB angle increased by 1.15° more in the higher overjet group but there was only weak evidence of an association (*P* = 0.086; 95% CI − 2.464 to 0.164).

**Conclusions:**

A slight straightening of the facial profile was observed in both groups with a statistically significant greater reduction in ANB arising in the group with larger baseline overjet. This translated into a marginal reduction in the overjet in this group.

## Background

An appreciation of changes in maxillo-mandibular relationships during the adolescent growth period is integral to planning the nature and timing of intervention, particularly growth modification directed at antero-posterior and vertical correction. Although a retrognathic mandible, prognathic maxilla or combination of both can contribute to increased overjet, McNamara (1981), in an analysis of 277 8- to 10-year-olds, observed a prognathic maxilla in only 25% of the class II sample with a retrognathic mandible predominating [1]. Similarly, Carter in a radiographic assessment of 30 untreated cases found a retrognathic mandible is typical [2], reflected clinically in a convex soft tissue profile [3].

It has been suggested that differences in craniofacial measures between class II and the class I malocclusion are established early in childhood and persist into the adult dentition [3–6] with growth trends in both groups of subjects being analogous [3–9]. However, reduced mandibular growth rates among class II subjects compared with normal controls have been observed throughout the circum-pubertal period [3, 10–12]. In contrast, no difference in mandibular growth in class II subjects have also been found from the deciduous dentition into the permanent dentition [3, 6]. Moreover, while no significant differences in the position of the maxillary base and dentition have been identified in class II subjects with growth [13, 14], other data describe significant increases in maxillary protrusion during the circum-pubertal period [5].

Based on further longitudinal data from growth studies, some straightening of the profile and reduction in facial convexity may occur during the pubertal growth phase [15], although this has not been a universal finding [12] and little change in the skeletal profile is thought to occur in late adolescence [8]. A greater increase in absolute mandibular length is intuitive as its overall dimension is greater than that of the maxilla with the percentage difference in the increase between mandibular and maxillary less significant; mandibular length also incorporates a marked vertical component, while maxillary growth is usually measured from the anterior nasal spine (ANS) to the posterior nasal spine (PNS) and is therefore essentially horizontal. Moreover, in an analysis of 8–18 year-olds with skeletal 2 patterns and increased overjet who had no orthodontic treatment, over 4mm more forward growth of the mandible relative to the maxilla was noted, although the occlusion and overjet was unaffected; this was attributed to the cuspal interdigitation [16]. Therefore, while increases in the absolute mandibular dimensions exceed those of the maxilla during adolescence, this does not normally result in occlusal improvement in class II malocclusion without active orthodontic intervention [17].

There is therefore conflicting data concerning growth patterns in class II and class I subjects with relatively little information pertaining to differences during the circum-pubertal period. The aim of this study was to assess differences in changes in maxillo-mandibular relationships during the adolescent growth period based on normal (< 4 mm) or increased (> 4 mm) overjet in childhood. We hypothesized that there is no difference in the changes in the A point, nasion, B point (ANB) angle during growth, between subjects presenting with normal and increased overjet in childhood. 

## Methods

The sample consisted of 65 subjects taken from the American Association of Orthodontists Foundation (AAOF) Craniofacial Growth Legacy Collections Project. Subjects were obtained from 7 out of 9 available collections based on the inclusion criteria within the primary studies. Specifically, 6 subjects were taken from the Burlington Growth Study, 8 from Denver Growth Study, 7 from Forsyth Twin Study, 5 from Iowa Growth Study, 12 from Mathews Growth Study, 25 from the Michigan Growth Study and 2 from the Oregon Growth Study.

Subjects were selected using a convenience sampling approach based on the following inclusion criteria: (1) lateral cephalograms available at ages 7–10 (T0) and 14–17 (T1) and (2) no prior orthodontic treatment. In order to obtain the largest possible sample and increase the statistical power, all eligibile subjects were included in the study. The subjects from Denver, Iowa and Oregon growth studies were of Caucasian origin, while those from Burlington, Mathews and Michigan growth studies were of predominantly Caucasian origin, although specific information was not available. Overall, the growth studies spanned a period from 1927 to 1967 with children having their first radiograph as early as 1 year of age.

**Fig. 1:**
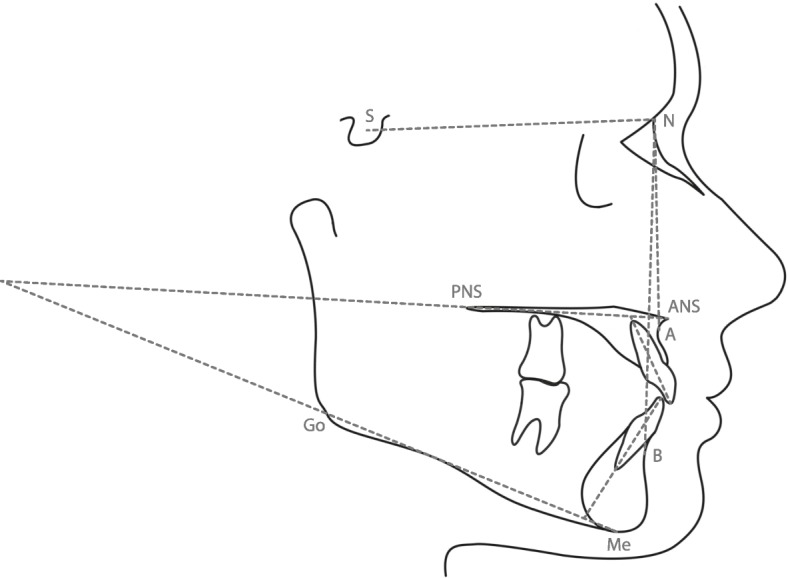
Cephalometric points, planes, lines and angles

Subjects were allocated into two groups based on baseline overjet as follows: Overjet in excess of 4 mm at age 7–10 (group 1) or ranging from 2 to 4 mm at age 7–10 (group 2). Where multiple cephalograms were available within each age group for a subject, the earliest film in the 7 to 10 years category and the latest film for the 14 to 17 years time-point were traced. Overjet measurements were obtained directly from data presented within the AAOF website. The cephalograms were traced by the same operator (M.T.) following training by the senior researcher (P.S.F.) using digital image analysis software (Digimizer Version 5.3.5). The following cephalometric angles were measured (Fig. 1): (1) SNA (sella, nasion, A point), (2) SNB (sella, nasion, B point), (3) ANB (A point, nasion, B point), (4) UIMx (long axis of upper incisor to maxillary plane), (5) LIMP (lower incisor to mandibular plane) and (6) MMPA (maxillary to mandibular plane). To determine intra-examiner reliability, 20 randomly chosen lateral cephalograms were retraced by the same examiner (M.T.) 2 weeks after the initial tracing using the same software to determine the error resulting from landmark selection or tracing. Reliability was assessed by using Bland-Altman’s limits of agreement for all measurements. It was found to be good for all parameters (Table 1).

**Table 1:** Assessment of the repeatability of measures in the study (*n* = 20)

	Mean	SD	95% CI
**SNA**			
Measurement 1	82.35	4.18	
Measurement 2	82.50	3.95	
Differences	0.15	0.49	− 0.08, 0.38
Average	82.43	4.11	
**SNB**			
Measurement 1	78.63	4.10	
Measurement 2	78.85	3.91	
Differences	0.22	0.54	− 0.03, 0.47
Average	78.74	4.01	
**ANB**			
Measurement 1	3.73	2.64	
Measurement 2	3.66	2.62	
Differences	− 0.07	0.24	− 0.18, 0.04
Average	3.69	2.63	
**MMPA**			
Measurement 1	25.00	4.12	
Measurement 2	24.85	3.98	
Differences	− 0.14	0.40	− 0.33, 0.04
Average	24.92	4.05	
**UIMx**			
Measurement 1	115.38	5.74	
Measurement 2	115.54	5.70	
Differences	0.16	0.42	− 0.04, 0.36
Average	115.46	5.72	
**LIMP**			
Measurement 1	96.85	3.99	
Measurement 2	96.68	4.14	
Differences	− 0.17	0.51	− 0.4, 0.07
Average	96.76	4.06	

Descriptive statistics were calculated for demographic and clinical data where available including linear and angular cephalometric measurements at baseline (7–10 years) and at the later period (14–17 years). The random effects linear regression models were fitted to account for multiple within patient measurements with dependent variables SNA, SNB and ANB angles and independent variables including overjet group, age and gender. All statistical analyses were conducted with STATA® version 16 software (Stata Corporation, College Station, TX, USA).

## Results

Overall, there were a similar number of males (*n* = 34; 52.3%) and females (*n* = 31; 47.7%). The two overjet groups at T0 (age 7–10 years) had a similar number of subjects with 33 subjects (50.8%) having an overjet above 4 mm (group 1) and 32 (49.2%) with an overjet of 2–4 mm (Table 2). Groups were comparable in terms of age at both time-points (Table 3).

**Table 2:** Sample demographics (*n* = 65)

	Group 1	Group2	Overall
	*Ν* = 32	*Ν* = 33	*Ν* = 65
**Age T0**			
Min–max	7–10	7–10	7–10
Mean (SD)	7.88 (0.98)	8.36 (0.9)	8.12 (0.96)
**Age T1**			
Min–max	14–17	14–17	14–17
Mean (SD)	15.84 (1.08)	15.85 (1.06)	15.85 (1.06)
**Sex**			
Male	15	19	34
Female	17	14	31

**Table 3:** Clinical and cephalometric descriptive results per overjet group at T0 and T1 (*n* = 65)

	At age 7–10 (T0)				At age 14–17 (T1)			
	Group 1		Group 2		Group 1		Group 2	
	Mean	SD	Mean	SD	Mean	SD	Mean	SD
Age	7.88	0.98	8.36	0.90	15.84	1.08	15.85	1.06
Overjet	6.21	1.86	2.98	0.54	5.16	1.58	3.28	1.33
SNA	81.25	2.92	80.04	3.80	81.84	3.46	81.40	3.52
SNB	75.85	2.28	77.20	3.03	78.35	3.08	79.46	3.41
ANB	5.42	2.16	3.08	1.91	3.51	1.93	1.96	1.83
MMPA	28.01	4.38	25.38	4.34	26.80	4.61	23.67	4.20
UIMx	113.97	4.83	115.92	4.86	114.36	5.99	113.75	5.31
LIMP	94.03	4.60	97.20	4.51	96.44	5.52	97.85	6.99

The mean SNA values were similar in both groups at baseline increasing from 0.59 to 1.36° in both groups (Table 3) with a slightly greater increase in the normal overjet group (group 2; Table 3). SNB values increased in both groups over the observation period (2.26 to 2.5°) with the mean SNB value being marginally higher in group 2 at the later time-point (79.46° vs. 78.35°). In terms of ANB, the mean ANB was higher at baseline in group 1 (5.42, SD 2.16°) than in group 2 (3.08, SD 1.91°). A difference remained at the later time-point, although this difference declined to 1.55° (Table 3).

The ANB angle reduced by 1.92° more in the higher overjet group compared to the 2–4 mm of overjet group with the association being statistically significant after taking age into account (*P* < 0.001; 95% CI 1.06 to 2.77; Table 4). As such, the profile straightened more in the higher overjet group. No significant gender-related effect (*P* = 0.624; 95% CI − 0.64 to 1.07) was observed. Random effects linear regression modelling revealed no significant increase in SNA angle in the > 4 mm overjet group compared to the 2–4 mm group after adjusting for age (0.94°, *P* = 0.22; 95% CI − 0.58 to 2.47). The SNB angle increased by 1.15° more in the higher overjet group after adjusting for age but there was only weak evidence of this association (*P* = 0.11; 95% CI − 2.38 to 0.24). When older, the adolescents had a 0.308 increase in the SNB angle compared to when they were younger (*P* < 0.001).

**Table 4:** Changes in mandibular and maxillary position (ANB, SNA and SNB) by overjet group (> 4 mm or 2-4 mm) at T0, adjusted for age and gender (*n* = 65)

Variable	Predictor		Β-Coefficient	95% CI	*P* value
ANB	Overjet group	2–4 mm	Reference		
		> 4 mm	1.92	1.06, 2.77	< 0.001
SNA	Overjet group	2–4 mm	Reference		
		> 4 mm	0.94	− 0.58, 2.47	0.22
SNB	Overjet group	2–4 mm	Reference		
		> 4 mm	− 1.07	− 2.38, 0.24	0.11

## Discussion

The results from the present analysis suggest that a reduction in facial convexity tends to occur with age irrespective of the start overjet, although this improvement may be marginally more marked in those with larger overjet in pre-adolescence. Specifically, inter-maxillary relationships appeared to change slightly in both groups with minor increases in SNA (0.59 to 1.36°) being dwarfed by more substantial increases in SNB (2.26 to 2.5°) leading to an overall reduction in facial convexity reflected in a decrease in ANB by 1.12 to 1.91°. This finding is in keeping with previous research [15, 17]. This improvement appears, however, to have marginal effect in terms of occlusal relationships with the overjet decreasing slightly more in the group experiencing more favourable growth.

The straightening of the profile observed over a mean observation of 7.7 years from 8.1 to 15.8 years reflects changes arising in juvenile, pre-pubertal and adolescent years. This finding mirrors a longitudinal study based on the Belfast Growth Study, in which the SNA angle did not change significantly between 5 and 15 years in all groups (classes I, II and III) while the SNB angle increased slightly in all groups except for the class II division 2 females [10]. Similarly, Chung and Wong [18] in an analysis of untreated skeletal class II males and females from ages 9 to 18 from the Bolton-Brush and Burlington Growth studies found that the SNA and SNB angles increased and the ANB angle decreased in all groups with age. The authors concluded that the skeletal class II relationship tended to improve with age, although no gender-related effect was observed in relation to angular measurements in keeping with the findings from the present study [18].

A demonstrable increase in SNB value (up to 2.5°) arose in both groups over the observation period. Subjects included in the present study were based on the initial overjet scores with growth in any direction therefore being conceivable. However, Riesmeijer (2004) in an analysis of 7- to 14- year-olds encompassing Fels, Michigan and Nijmegan databases, observed a greater increase in SNB in the class I group. Similarly, the ANB angle reduced less with age in both genders in the class II compared with the class I group [19]. The latter was confirmed by Lundström and Woodside who concluded that mandibular retrognathia can be determined at age 9 becoming more marked with age [20]. Conversely, in a longitudinal study involving 30 participants between 12 and 17 years of age, a 1° reduction in ANB angle was found in class II males while a more backwards and downward mandibular growth pattern was apparent in females [2]. No gender-related trends were observed in the present larger sample suggesting that antero-posterior skeletal and occlusal changes are likely to be similar among both males and females during juvenile and adolescent growth.

Mean overjet was observed to reduce slightly in group 1. This may well reflect improvement in the inter-maxillary relationships; however, the difference was limited (1.05 mm). Conversely, a marginal increase in overjet (0.3 mm) was identified in group 2. Importantly, however, the overjet at the later time-period remained normal (3.28 mm, SD 1.33) in the latter cohort, while continuing to be excessive in group 1 (5.16 mm, SD 1.58). Similarly, in an analysis of untreated skeletal class I subjects from the Bolton-Brush and Burlington Growth Studies, Chung and Mongiovi observed that overjet did not worsen with age [21]. The clinical relevance of this is clear with intervention required to address class II malocclusion, while early achievement of a class I incisor relationship is likely to be relatively stable over a period of growth. The latter has variously been confirmed with antero-posterior correction among the more stable orthodontic changes [22, 23].

In terms of dental change, marginal improvement was observed in the present study in the group with larger initial overjet. This contrasts with a previous analysis of 25 untreated subjects with class II malocclusion observed for a 2.5-year period from the deciduous into the mixed dentition, in which the clinical signs of class II malocclusions in the deciduous dentition persisted or become exaggerated. However, the cephalometric changes of the class II sample over this growth period showed significantly greater growth increments in the maxilla and smaller growth increments in the mandible [5]. Conversely, Bishara et al. [6], in a comparison of untreated class II division I subjects with normal subjects in the mixed early permanent dentition, found that class II subjects with increased ANB angle had similar growth profiles to those of normal subjects. However, the analysis in this study did not extend beyond an average age of 12.2 years, when active growth is still ongoing. The authors concluded that class II malocclusion is not ‘self-correcting’ in growing patients [6]. It is noteworthy that the assessment undertaken in the present analysis was more sustained incorporating both the period of maximal pre-pubertal growth and adolescent growth.

In terms of limitations, the present study was retrospective being based on historical data made available as part of the AAOF Craniofacial Growth Legacy Collections Project. As such, there are constraints in relation to the generalizability of the findings with the majority of subjects being white Caucasian. Moreover, the historical nature of the data is potentially problematic in view of the possibility of secular trends relating to changing facial appearance. The latter is a particular problem in comparative studies using historical controls in conjunction with contemporary groups [24]. Notwithstanding this, while subtle changes in craniofacial form have been attributed to a ‘year of birth’ effect [25], there is no evidence to suggest that growth patterns over the adolescent period have changed over the past 90 years. Analysis was limited to cephalometric data; as such, there are associated constraints in terms of reliability and validity [26]; notwithstanding this, in view of the historical nature of the data, clinical correlation was not possible. Notwithstanding this, serial cephalometric analysis continues to form the mainstay for assessing the pattern and magnitude of craniofacial growth.

## Conclusions

A reduction in facial convexity characterized by a decrease in ANB angle was observed in both groups with a significantly greater reduction in ANB arising in the group with larger baseline overjet. This change translated into a marginal improvement in the overjet relative to the group with normal initial overjet.

## Data Availability

The datasets used and/or analysed during the current study are available from the corresponding author on reasonable request.

## Abbreviations

AAOF: American Association of Orthodontists Foundation
ANB: A point, nasion, B point
ANS: Anterior nasal spine
CI: Confidence interval
LIMP: Lower incisor to mandibular plane angle
MMPA: Maxillary to mandibular plane angle
*P*: *P* value
PNS: Posterior nasal spine
SD: Standard deviation
SNA: Sella, nasion, A point angle
SNB: Sella, nasion, B point
UIMx: Long axis of upper incisor to maxillary plane angle

## References

[1] McNamara JA (1981). Components of class II malocclusion in children 8-10 years of age. Angle Orthodon.

[2] Carter NE (1987). Dentofacial changes in untreated class II division 1 subjects. Br J Orthod.

[3] Bishara SE (1998). Mandibular changes in persons with untreated and treated class II division 1 malocclusion. Am J Orthod Dentofacial Orthop.

[4] Buschang PH, Tanguay R, Demirjian A, LaPalme L, Turkewicz J (1988). Mathematical models of longitudinal mandibular growth for children with normal and untreated class II, division 1 malocclusion. Eur J Orthod.

[5] Baccetti T, Franchi L, McNamara JA, Tollaro I (1997). Early dentofacial features of class II malocclusion: a longitudinal study from the deciduous through the mixed dentition. Am J Orthod Dentofacial Orthop.

[6] Bishara SE, Jakobsen JR, Vorhies B, Bayati P (1997). Changes in dentofacial structures in untreated class II division 1 and normal subjects: a longitudinal study. Angle Orthod.

[7] Vásquez, M.J., Baccetti, T., Franchi, L., McNamara, J.A., 2009. Dentofacial features of class II malocclusion associated with maxillary skeletal protrusion: a longitudinal study at the circumpubertal growth period. Am J Orthod Dentofacial Orthop 135, 568.e1–7; discussion 568-569. 10.1016/j.ajodo.2007.05.026. 10.1016/j.ajodo.2007.05.026 19409335

[8] Baccetti T, Stahl F, McNamara JA (2009). Dentofacial growth changes in subjects with untreated class II malocclusion from late puberty through young adulthood. Am J Orthod Dentofacial Orthop..

[9] Yoon SS, Chung C-H (2015). Comparison of craniofacial growth of untreated Class I and class II girls from ages 9 to 18 years: a longitudinal study. Am J Orthod Dentofacial Orthop.

[10] Kerr WJ, Hirst D (1987). Craniofacial characteristics of subjects with normal and postnormal occlusions—a longitudinal study. Am J Orthod Dentofacial Orthop.

[11] Ngan PW, Byczek E, Scheick J (1997). Longitudinal evaluation of growth changes in class II division 1 subjects. Semin Orthod.

[12] Stahl F, Baccetti T, Franchi L, McNamara JA (2008). Longitudinal growth changes in untreated subjects with class II division 1 malocclusion. Am J Orthod Dentofacial Orthop.

[13] Anderson DL, Popovich F (1983). Lower cranial height vs craniofacial dimensions in angle class II malocclusion. Angle Orthod.

[14] Varrela J (1998). Early developmental traits in class II malocclusion. Acta Odontol Scand.

[15] Lande MJ (1952). Growth behavior of the human bony facial profile as revealed by serial cephalometric roentgenology 1. Angle Orthod..

[16] You ZH, Fishman LS, Rosenblum RE, Subtelny JD (2001). Dentoalveolar changes related to mandibular forward growth in untreated Class II persons. Am J Orthod Dentofacial Orthop..

[17] Lux CJ, Burden D, Conradt C, Komposch G (2005). Age-related changes in sagittal relationship between the maxilla and mandible. Eur J Orthod..

[18] Chung CH, Wong WW (2002). Craniofacial growth in untreated class II subjects: a longitudinal study. Am J Orthod Dentofacial Orthop.

[19] Riesmeijer AM, Prahl-Andersen B, Mascarenhas AK, Joo BH, Vig KWL (2004). A comparison of craniofacial class I and class II growth patterns. Am J Orthod Dentofacial Orthop.

[20] Lundström A, Woodside DG (1983). Longitudinal changes in facial type in cases with vertical and horizontal mandibular growth directions. Eur J Orthod..

[21] Chung CH, Mongiovi VD (2003). Craniofacial growth in untreated skeletal class I subjects with low, average, and high MP-SN angles: a longitudinal study. Am J Orthod Dentofacial Orthop.

[22] Fidler BC, Artun J, Joondeph DR, Little RM (1995). Long-term stability of angle class II, division 1 malocclusions with successful occlusal results at end of active treatment. Am J Orthod Dentofacial Orthop.

[23] Francisconi MF, Henriques JF, Janson G, Freitas KM, Santos PB (2013). Stability of class II treatment with the bionator followed by fixed appliances. J Appl Oral Sci.

[24] Papageorgiou SN, Koretsi V, Jäger A (2017). Bias from historical control groups used in orthodontic research: a meta-epidemiological study. Eur J Orthod.

[25] Antoun JS, Cameron C, Sew Hoy W, Herbison P, Farella M (2015). Evidence of secular trends in a collection of historical craniofacial growth studies. Eur J Orthod.

[26] Baumrind S, Frantz RC (1971). The reliability of head film measurements. 2. Conventional angular and linear measures. Am J Orthod..

